# Optimal dietary vitamin B1 content enhanced egg production, eggshell thickness, and serum antioxidant status in breeder geese

**DOI:** 10.5713/ab.24.0751

**Published:** 2025-02-27

**Authors:** Lin Dai, Baowei Wang, Qian Li, Mingai Zhang, Jing Zhang, Bin Yue, Min Kong, Binghan Wang, Wenlei Fan

**Affiliations:** 1 College of Animal Science and Technology, Qingdao Agricultural University, Qingdao, China; 2 College of Food Science and Engineering, Qingdao Agricultural University, Qingdao, China; 3 College of Science and Information Science, Qingdao Agricultural University, Qingdao, China; 4 Institute of High-Quality Waterfowl, Qingdao Agricultural University, Qingdao, China; 5 Qingdao Huihe Biotechnology Co., Ltd., Qingdao, China

**Keywords:** Antioxidation, Egg Production, Geese, Nutrient Digestion, Reproduction, Vitamin Requirement

## Abstract

**Objective:**

This study aimed to evaluate the effects of vitamin B1 (VB1) supplementation on laying performance, egg quality, serum biochemical parameters, antioxidant status, and nutrient digestion in breeder geese.

**Methods:**

A total of 150 geese (30 males and 120 females, aged 34 weeks) were randomly assigned to 6 dietary treatment groups, each 5 replicates of 5 birds (1 male and 4 females). The geese were fed a basal diet supplemented with 0 (control), 1, 2, 3, 4, or 5 mg/kg of VB1 for 10 weeks.

**Results:**

VB1 supplementation had no significant effects on average feed intake, average egg weight, feed-to-egg ratio, egg shape index, eggshell strength, protein height, and Haugh unit (p>0.05). However, it increased egg-laying rate, eggshell thickness, and yolk color (p<0.05) in a quadratic manner, with the maximum values observed at 2 mg/kg VB1. Supplementing 2 mg/kg VB1 reduced serum aspartate transaminase activity (p<0.05), but did not affect serum alanine transaminase activity, lipid and protein concentrations (p>0.05). Serum glutathione peroxidase and total superoxide dismutase activities were enhanced by VB1 supplementation (p<0.05), while total antioxidant capacity and malondialdehyde concentration remained unchanged (p>0.05). Additionally, VB1 supplementation at 2 mg/kg increased crude ash digestibility, but did not affect the digestibility of ether extract, crude protein, calcium, and phosphorus.

**Conclusion:**

Dietary supplementing VB1 improved egg-laying performance, egg quality, antioxidant status, and mineral absorption in breeder geese. The optimal dietary VB1 concentration ranged from 3.83 to 4.81 mg/kg for improving egg production and quality, while higher concentrations of 4.99 to 6.51 mg/kg were needed to boost serum antioxidant status.

## INTRODUCTION

Geese (*Anas cygnoides*) are among the most economically important farm birds, with over 600 million reared commercially in China. However, their relatively low laying performance, with an egg-laying rate (LR) of less than 50% during the breeding season, poses a significance challenge to the growth of the goose industry [1–3]. This limitation has driven increasing interest in strategies to improve laying performance, with a particular focus on developing nutritionally balanced feed formulation.

Vitamin B1 (thiamine, VB1) is essential for energy metabolism, neurological function, antioxidant defense, digestion, and cardiovascular health. Dietary VB1 levels have been shown to influence the development and health of various animal species [4]. VB1 deficiency can cause memory and learning impairments, immune suppression, blood-brain barrier dysfunction, and neurological disorders in animals [5–9]. In rats, VB1 deficiency has been linked to delayed sexual maturation, reduced reproductive capacity, and lactation failure [10]. In livestock, VB1 supplementation has been associated with improved reproductive performance. For example, supplementation of VB1, vitamin E, and selenium improved pregnancy rates in Merino ewes [11], highlighting VB1’s importance in reproductive system function. In chickens, appropriate levels of VB1 supplementation enhanced productive and slaughter performances [12]. Chen et al [13] demonstrated that a corn-soybean meal based diet sufficiently met the VB1 requirements of Longyan egg-laying ducks from 22 to 42 weeks of age. Moreover, our previous research determined the dietary VB1 requirements for growing geese: 5.60 mg/kg of feed for geese aged 0 to 4 weeks and 4.98 mg/kg for those aged 5 to 15 weeks [14].

Research on the dietary VB1 requirements for laying geese remains limited. In the absence of specific data, the Dale N [15] recommends using the requirements established for growing geese (2.0 mg/kg) and adult geese (1.8 mg/kg), with breeding geese often using nutrient estimates for chickens. To fill this knowledge gap, we supplemented diets with graded levels of VB1 and assessed its effects on laying performance, egg quality, serum biochemical parameters, antioxidant status, and nutrient digestion in laying Wulong geese. This study aims to determine the optimal dietary VB1 dosage for breeding geese, providing evidence-based recommendations for feed supplementation during the laying period.

## MATERIALS AND METHODS

### Birds, Management and Diet

The experiment was approved by the Animal Ethics Committee of Qingdao Agricultural University (IACUC, approval number: QAU20210319), in accordance with the Guidelines for Experimental Animals established by the Ministry of Science and Technology. A total of 150 Wulong breeder geese (30 males and 120 females), aged 34 weeks with an average body weight of 3±0.2 kg (mean±standard deviation), were obtained from the Breeding Center of Qingdao Agricultural University (Shandong, China), where the experiment was conducted. The geese were randomly assigned to 6 dietary treatments in a randomized complete block design, with egg production level as a blocking factor, using the Experimental Animal Allocation Procedure (EAAP). Geese were reared in a free-range system within fenced pens, with half of each pen covered by a shed containing feeders and cylindrical plastic water tanks. Each pen measured 12 m×1.24 m and was equipped with a wire floor inside the shed and a sand floor in the open yard. Geese were randomly allocated to 30 pens, each had 5 birds (1 male and 4 female). During the experimental period, geese had *ad libitum* access to feed and water and were housed outdoors under natural daylight conditions. Birds were fed twice daily, at 08:00 and 17:00. The basal diet was formulated according to Dale [15] recommendations to meet the nutrient requirements of breeder geese. The ingredient composition and nutrient content of the diet are presented in Table 1, with an analyzed VB1 level of 2.26 mg/kg.

### Experimental design

The 30 pens of geese were randomly assigned to 6 dietary treatment groups, each with 5 replicate pens, and randomly allocated to the diets supplemented with 0 (control), 1, 2, 3, 4, or 5 mg VB1 per kg of diet. Each pen, containing 5 geese, served as a biologically experimental unit to minimize confounding factors such as social interactions, feed competition, and environmental influences on individual performance [16,17]. The experiment lasted for 10 weeks. This design was practically manageable, ensuring consistent experimental conditions (e.g., diet preparation, monitoring, and data collection) without overburdening resources.

Crystalline VB1 (purity 98%), purchased from Qingdao Puxing Biotechnology Co., Ltd. (Qingdao, China), was mixed into the vitamin premix and then incorporated into the final diet.

### Laying performance and collection of blood sample

The feed consumption of birds in each pen was recorded daily to calculate the average daily feed intake (ADFI), average egg weight (AEW), LR, and feed-to-egg ratio (F/E). On the last day of the experiment, 2 female geese from each pen were randomly selected, fasted for 12 h, and had blood samples collected from the wing vein into coagulant tubes. The samples were centrifuged at 1,520×g at 4°C for 10 min to separate the serum, which was then stored at −20°C for analysis.

### Egg quality

Eggs laid during the last two weeks of the experiment were collected daily and stored at 16°C with 75% relative humidity. The egg quality analyses included egg weight, shape index, eggshell strength, eggshell thickness, albumen height, yolk color, Haugh unit, and yolk weight. All analyses were performed within 24 hours of collection. Egg length and width were measured using a digital caliper, and the egg shape index (ESI, %) was calculated as width/length. Yolk weight was expressed as a percentage of total egg weight. Eggshell thickness was measured at the two poles and the middle of the shell with a digital micrometer. The remaining analyses were performed using an Egg Analyzer (DET-6000; NABEL Co., Ltd., Kyoto, Japan) according to the manufacturer’s protocols. Detailed procedures are provided in the Supplement 1.

### Measurement of biochemical parameters and antioxidant indices

Serum samples were analyzed for concentrations of triglyceride (TG), total cholesterol (T-CHO), albumin (ALB), total protein (TP), and malondialdehyde (MDA), the activity of aspartate transaminase (AST), alanine transaminase (ALT), glutathione peroxidase (GSH-Px), total superoxide dismutase (T-SOD), as well as total antioxidant capacity (T-AOC). All analyses were performed using commercial kits (Nanjing Jiangcheng Bioengineering Institute, Nanjing, China) according to the manufacturer’s protocols. Detailed protocols are described in the Supplement 1.

### Nutrient utilization

In the final week of the experiment, two female geese from each replicate pen were randomly selected and placed in individual metabolic cages. After a 24-hour fast, each bird was fed 120 g/day of the diet for 3 days. Fecal excreta were collected daily for 3 days, pooled by cage, and dried at 65°C to a consist weight. Both feed and fecal excreta samples were ground and analyzed for crude fat (ether extract), crude protein, crude ash, calcium (Ca), and phosphorus (P) following AOAC methods [18]. Ca and P contents were determined using inductively coupled plasma-optical emission spectrometry (Optima 8×00; PerkinElmer Inc., Alpharetta, GA, USA).

### Statistical analysis

The effects of dietary VB1 supplementation were analyzed using one-way ANOVA in SPSS statistical software (version 20.0, SPSS, Chicago, IL, USA). For variables showing significant differences, Duncan’s multiple-range test was used to compare means between treatments. Polynomial contrasts were applied to evaluate linear and quadratic effects of VB1 levels. Data are presented as means with pooled standard error of the mean, and differences were considered statistically significant at p<0.05.

The estimation of the maximum responses to total dietary VB1 was conducted using a quadratic polynomial model [19,20] as follows:


$$\text{Y}=\beta_{0}+\beta_{1}\times \text{X}+\beta_{2}\times {\text{X}}^{2},$$

where Y is the responsive variable, X represents dietary total VB1 concentration (mg/kg diet); β_0_ is the intercept; β_1_ stands for the linear coefficient; and β_2_ as the quadratic coefficient. The total dietary VB1 concentration corresponding to the maximum response is calculated as VB1 = − *β_1_*÷(2×*β_2_*).

## RESULTS

### Egg-laying performance

**Figure 1:**
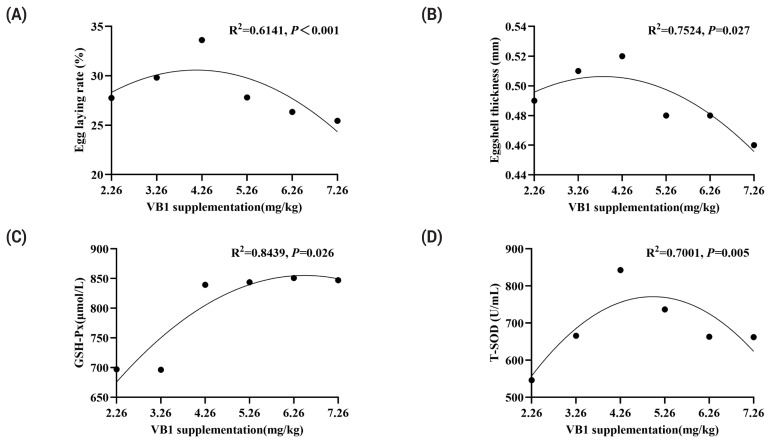
The effect of supplementing vitamin B1 in the diet on egg production rate (A), eggshell thickness (B), GSH-Px(C), and T-SOD (D). GSH-Px, glutathione peroxidase; T-SOD, total superoxide dismutase.

As shown in Table 2, the VB1 level in the diet had a significant effect on LR (p<0.05), exhibiting a quadratic response (p< 0.05). The maximum LR was achieved at 2 mg/kg VB1 supplementation (Figure 1A), corresponding to a total of 4.14 mg/kg VB1 in the diet (Figure 1A). In contrast, there was no significant effect of VB1 supplementation on ADFI, AEW, and F/E (p>0.05).

### Egg quality

Egg quality results are presented Table 3. Dietary VB1 supplementation had significant effects on eggshell thickness (Figure 1B) (p<0.05) and egg yolk color (p<0.05), with a quadratic responsive fashion (p<0.05). The maximum value of eggshell thickness was reached at 2 mg/kg VB1 supplementation, corresponding to a total of 3.83 mg/kg VB1 in the diet. VB1 supplementation also had a significant effect on the egg yolk percentage (p<0.05). However, it had no significant effects on the egg weight, ESI, shell strength, protein height, and Haugh units (p>0.05).

### Serum biochemical parameters

As shown in Table 4, dietary VB1 supplementation at a dose of 2 mg/kg significantly reduced serum aspartate transaminase activity (p<0.05). In contrast, serum concentrations of TGs, T-CHO, ALB, TP, and ALT activity were not affected by dietary VB1 supplementation (p>0.05).

### Antioxidant indicators

Serum antioxidant indicators are shown in Table 5. Dietary VB1 supplementation significantly enhanced the activity of GSH-Px (p<0.05) and T-SOD (p<0.05) in a quadratic response pattern (Figures 1C, 1D). The maximum GSH-Px activity occurred at 4 mg/kg VB1 supplementation, the corresponding to a total dietary VB1 concentration of 6.51 mg/kg, while the highest T-SOD activity was observed at 2 mg/kg VB1 supplementation, corresponding to 4.99 mg/kg VB1 in the diet. However, dietary VB1 supplementation had no significant effects on T-AOC activity and MDA concentration (p>0.05).

### Nutrient digestibility

Table 6 shows that dietary VB1 supplementation increased crude ash digestibility (p<0.05) in a quadratic response pattern, with the highest digestibility observed at a dose of 2 mg/kg. However, it had no significant effects on digestibility of ether extract, crude protein, calcium, and phosphorus (p> 0.05).

### Optimal dietary vitamin B1 concentration

Several measures showed a quadratic relationship with dietary VB1 concentration; therefore, quadratic polynomial analysis was performed to determine the optimal VB1 concentration associated with the maximum response for each measure. The results are presented in Table 7. The optimal total VB1 concentration in the diet was 4.14 mg/kg LR, 3.83 mg/kg for eggshell thickness, 4.81 mg/kg for yolk color, 4.36 mg/kg for egg yolk percentage, 6.51 mg/kg for GSH-Px activity, and 4.99 mg/kg for T-SOD activity, respectively.

## DISCUSSION

VB1 supports poultry growth, energy metabolism, and offspring health [13,14,21]. Both deficiency and excess of VB1 can reduce productivity and cause metabolic disturbances, illness, and even mortality [22]. Research in ducks and chickens has highlighted the role of VB1 in improving production performance [23,24]. Our previous studies have shown its positive effects on the reproductive performance of geese [25]. In this study, we demonstrated its effects on breeder geese. We found dietary supplementation of 2 mg/kg of VB1 significantly enhanced LR in breeder geese. Previous studies found that VB1 deficiency can impair muscle function, leading to reduced egg production in poultry [26–28]. However, excessive VB1 supplementation also negatively affected egg production, as observed in the present study. These results are consistent with research on other B vitamins, such as riboflavin (B2) and pyridoxine (B6), which are crucial for poultry health [29,30]. Therefore, optimizing vitamin supplementation in poultry diets is essential for meeting nutritional requirements, improving production performance, and enhancing economic efficiency.

This study demonstrated that VB1 supplementation improved egg quality, such as increased eggshell thickness and yolk color, with the most pronounced effect on yolk pigmentation observed at a dose of 2 mg/kg. Conversely, excessive VB1 intake reduced eggshell thickness. Increased eggshell thickness is critical for minimizing egg breakage and embryonic mortality by preventing water loss, heat stress, and microbial invasion during incubation [31]. Previous studies have shown that varying dietary levels of VB1 can improve egg quality in breeder ducks [13]. Additionally, optimizing B-vitamin combinations has been shown to enhance overall egg quality [32], which aligns with our findings in this study. Comparisons with other vitamins, such as vitamin A and vitamin G, underscore the importance of a balanced intake of B vitamins is vital for maximizing egg quality and production efficiency in poultry [33]. While few studies have specifically address VB1’s role in egg quality, the broader importance of vitamin balance in maintaining egg quality is widely recognized [34–36] Further research is necessary to elucidate the specific mechanisms by which vitamins influence egg quality characteristics across different poultry species.

Serum biochemical parameters are significant indicators of the nutritional and health status of geese. This study found that VB1 supplementation had no significant effect on blood lipids and proteins, but it effectively regulated liver enzyme activities by reducing AST activity. Our results are align with previous studies in chickens [37]. In ducks, VB1 supplementation moderately affects blood protein levels, primarily due to its involvement in energy metabolism rather than direct regulation of protein synthesis [38]. In contrast, VB2, VB6, and VB12 exert more pronounced effects on lipid and protein metabolism, as they are more directly involved in coenzyme functions related to amino acid and fatty acid metabolism [39]. However, VB1 is primarily involved in energy production, making it less directly associated with lipid and protein regulation. Elevated AST activity is commonly linked to liver damage [40]. Studies in rats have demonstrated that VB1 deficiency negatively affects AST activity, disrupting energy metabolism and overall health [41–43]. Based on these findings, appropriate VB1 supplementation could improve liver health in geese without significant effects on its lipid and protein dynamics.

Antioxidant enzymes play a crucial role in mitigating oxidative stress, which can impair health and productivity in animals. This study demonstrated that appropriate VB1 supplementation enhanced serum GSH-Px and T-SOD activity in breeder geese, thereby enhancing their antioxidant status. VB1 is essential for the biosynthesis of antioxidant compounds [44], supporting overall antioxidant function in ducks [36], and enhancing antioxidant enzyme activity in fish by regulating cellular redox balance [45]. Ma et al. [46] found that supplementation of 200 mg/kg VB1 in a high-concentrate diet improved the total plasma antioxidant capacity in goats. These findings indicate that VB1 likely enhances antioxidant enzyme activity in geese by promoting the biosynthesis of antioxidant substances. Furthermore, studies on vitamins VB9 and VB12 suggest that combining these vitamins can have synergistic effects on improving egg production and overall health in poultry [47]. The commercial implications of these findings suggest that B-vitamins supplementation can reduce oxidative damage and optimize poultry health under farm conditions. However, a limitation of this study is its focus on geese, warranting further investigation across other poultry species to better understand the broader applicability of these findings.

The enhanced production performance of breeder geese may be resulted from improved nutrient digestion. In this study, dietary supplementation with 2 mg/kg of VB1 increased crude ash digestibility, indicating enhanced mineral absorption. Crude ash content, which reflects the total mineral content in poultry manure, serves as an indicator of mineral digestion and utilization [48]. Mohseni et al. [49] reported that dietary VB1 supplementation increased total body ash content in juvenile fish, suggesting improved mineral utilization efficiency. Similarly, VB1 levels were found to indirectly enhance nutrient utilization efficiency in broiler chickens [50]. VB1 interacts with proteins through non-coenzyme mechanisms, potentially modulating mineral utilization [8,51,52]. Research on other B vitamins, such as B2 and B6, further emphasizes their roles in improving mineral absorption and utilization in poultry [24,53]. These findings underscore the critical role of B-vitamins in optimizing mineral absorption across poultry species. However, limitations of this study include the absence of long-term performance data and the influence of varying environmental conditions, which may affect results. Future research should address these factors to enhance the practical application of vitamin supplementation in commercial poultry production.

## CONCLUSION

This study demonstrates that VB1 supplementation enhanced egg-laying performance, egg quality, antioxidant status, and mineral absorption in breeder geese. The optimal dietary total VB1 concentration ranged from 3.83 to 4.81 mg/kg for improving egg production and quality, while higher concentrations of 4.99 to 6.51 mg/kg were required to boost serum antioxidant status. Future research should explore the long-term effects of VB1 on reproduction and overall health in geese, as well as investigate potential interactions between VB1 and other vitamins to optimize poultry productivity and health.

## Figures and Tables

**Table 1:** Composition and nutrient concentrations of the basal diet

Items	Dry matter basis (%)
Ingredients	
Corn	60.00
Soybean meal	23.50
Wheat bran	3.00
Chrysanthemum pedicel powder	3.00
Soybean oil	2.00
Limestone	6.00
Ca (HCO_3_)_2_	1.50
NaCl	0.30
Trace elements premix^1)^	0.50
Vitamin premix^1)^	0.10
Methionine	0.10
Nutrient concentrtions^2)^	
ME (MJ/Kg)	11.48
CP	16.22
CF	4.05
Ca	2.64
P	0.35
Lys	0.67
Met + Cys	0.57
Trp	0.19
Thr	0.61
Cl	0.14
VB_1_ (mg/kg)	2.26

1) The multi-vitamin and trace elements provided nutrients per kg of diets: vitamin A 9,000 IU, vitamin D_3_ 2,000 IU, vitamin E 40 mg, vitamin K_3_ 0.8 mg, vitamin B_2_ 4.0 mg, nicotinic acid 30 mg, pantothenate 11 mg, vitamin B6 4.0 mg, biotin 0.2 mg, folic acid 0.5 mg, vitamin B_12_ (1%) 12 ug, selenium 0.5 mg, iron 80 mg, manganese 30 mg, copper 4 mg, iodine 0.3 mg, zinc 0.3 mg.

2) CP, CF, Ca, P, Cl, and VB1 are analyzed values, determined with methods in accordance with the Chinese National Standard [54]. The ME, Lys, Met+Cys, and Thr contents are calculated values.

ME, metabolizable energy; CP, crude protein; CF, crude fiber; Lys, lysine; Met, methionine; Cys, cysteine; Trp, tryptophane; Thr, threonine; Cl, chlorine; VB1, vitamin B1.

**Table 2:** Effects of dietary vitamin B1 supplementation on production performance indexes in breeder geese

Items	Treatments^1)^ (VB1 mg/kg)						SEM	p-value		
	
	0 (CON)	1	2	3	4	5		ANOVA	Linear	Quadratic
ADFI (g/d)	181.44	181.63	193.29	183.36	184.22	175.06	2.57	0.521	0.529	0.137
AEW (g)	122.89	126.27	128.03	126.30	125.53	125.68	0.81	0.650	0.567	0.173
F/E	5.59	5.15	5.09	5.49	5.91	5.83	0.14	0.456	0.190	0.250
LR (%)	27.76^bc^	29.82^b^	33.62^a^	27.81^bc^	26.34^c^	25.44^c^	0.59	<0.001	0.001	<0.001

1) Data represent the means of 5 replicate pens per treatment.

a–c Means within the same row with different superscripts differ significantly (p<0.05).

SEM, standard error of the mean; ADFI, average daily feed intake; AEW, average egg weight; F/E, feed-to-egg ratio; LR, egg-laying rate.

**Table 3:** Effects of vitamin B1 supplementation on egg quality of breeder geese

Items	Treatments^1)^ (VB1 mg/kg)						SEM	p-value		
	
	0 (CON)	1	2	3	4	5		ANOVA	Linear	Quadratic
Egg weight (g)	119.72	130.76	132.10	130.17	122.45	120.78	1.70	0.090	0.504	0.008
Egg shape index	1.45	1.44	1.43	1.44	1.43	1.43	0.00	0.600	0.109	0.917
Eggshell strength (kg)	4.95	4.95	4.96	4.95	4.96	4.93	0.00	0.625	0.387	0.290
Eggshell thickness (mm)	0.49^abc^	0.51^ab^	0.52^a^	0.48^bc^	0.48^bc^	0.46^c^	0.01	0.006	0.004	0.027
Protein height (mm)	15.19	14.61	14.63	14.57	15.46	14.87	0.12	0.183	0.714	0.198
Yolk color	3.13^c^	3.53^ab^	4.11^a^	3.65^ab^	3.62^ab^	3.24^bc^	0.08	0.002	0.370	0.006
Haugh Unit	118.01	117.67	118.52	117.20	118.26	117.53	0.18	0.304	0.593	0.776
Egg yolk percentage	33.30^abc^	35.59^ab^	36.43^a^	35.80^ab^	32.66^bc^	32.28^c^	0.47	0.016	0.084	0.003

a–c Means within the same row with different superscripts differ significantly (p<0.05).

SEM, standard error of the mean.

**Table 4:** Effects of vitamin B1 supplementation on serum biochemical indicators in breeder geese

Items	Treatments^1)^ (VB1 mg/kg)						SEM	p-value		
	
	0 (CON)	1	2	3	4	5		ANOVA	Linear	Quadratic
TG (mmol/L)	2.34	2.50	2.59	2.83	2.57	1.30	0.23	0.499	0.330	0.115
T-CHO (mmol/L)	3.63	3.76	4.28	4.30	4.22	3.60	0.27	0.949	0.838	0.355
ALB (g/L)	36.70	37.35	37.49	38.26	37.17	38.44	0.70	0.985	0.574	0.915
TP (g/L)	52.69	56.30	63.07	56.83	54.04	57.66	2.03	0.790	0.788	0.433
AST (U/L)	17.73^a^	14.98^ab^	13.49^b^	16.63^ab^	15.28^ab^	15.68^ab^	0.41	0.047	0.409	0.057
ALT (U/L)	10.60	10.81	8.09	9.68	9.48	9.24	0.48	0.660	0.376	0.490

1) Data represent the means of 10 geese per treatment.

a,b Means within the same row with different superscripts differ significantly (p<0.05).

SEM, standard error of the mean; TG, triglycerides; T-CHO, total cholesterol; ALB, albumin; TP, total protein; AST, aspartate aminotransferase; ALT, alanine aminotransferase.

**Table 5:** Effects of vitamin B1 supplementation on antioxidant indicators in serum of breeding female geese

Items	Treatments^1)^ (VB1 mg/kg)						SEM	p-value		
	
	0 (CON)	1	2	3	4	5		ANOVA	Linear	Quadratic
T-AOC (mg/mL)	0.31	0.29	0.41	0.38	0.36	0.31	0.18	0.309	0.592	0.072
GSH-Px (μmol/L)	697.28^b^	696.27^b^	839.39^a^	843.76^a^	850.95^a^	847.17^a^	29.27	0.034	0.037	0.026
T-SOD (U/mL)	546.32^b^	665.83^ab^	842.88^a^	736.71^ab^	663.10^ab^	662.25^ab^	26.65	0.028	0.310	0.005
MDA (nmol/mL)	3.28	3.06	1.79	3.15	3.66	4.17	0.25	0.125	0.123	0.051

1) Data represent the means of 10 geese per treatment.

a,b Means within the same row with different superscripts differ significantly (p<0.05).

SEM, standard error of the mean; T-AOC, total antioxidant capacity; GSH-Px, glutathione peroxidase; T-SOD, total superoxide dismutase; MDA, malondialdehyde.

**Table 6:** Effect of vitamin VB1 supplementation on nutrient utilization efficiency of breeder geese

Items	Treatments^1)^ (VB1 mg/kg)						SEM	p-value		
	
	0 (CON)	1	2	3	4	5		ANOVA	Linear	Quadratic
Ether extract (%)	74.93	75.21	78.58	80.32	75.80	76.47	0.74	0.228	0.448	0.076
Crude protein (%)	71.68	76.15	84.09	82.73	74.06	78.60	1.48	0.090	0.337	0.039
Crude ash (%)	18.35^c^	21.39^bc^	34.48^a^	28.96^ab^	29.98^ab^	23.21^bc^	1.38	0.001	0.042	0.001
Calcium (%)	20.32	24.20	26.54	24.31	22.57	21.59	0.96	0.519	0.969	0.076
Phosphorus (%)	49.28	48.57	49.47	53.66	44.58	49.83	1.46	0.594	0.844	0.754

1) Data represent the means of 10 geese per treatment.

a–c Means within the same row with different superscripts differ significantly (p<0.05).

**Table 7:** Quadratic polynomial^1)^ response regressions with total dietary VB1 concentration (mg/kg diet) in breeder geese

Parameters	Regression equation	VB1 for maximum response (mg/kg)	p-value	R^2^
Egg laying rate	Y = −0.6407X^2^+5.304X+19.60	4.14	0.001	0.6141
Eggshell thickness	Y = −0.004286X^2^+0.0328X+0.4435	3.83	0.027	0.7524
Yolk color	Y = −0.1132X^2^+1.088X+1.263	4.81	0.006	0.7988
Egg yolk percentage	Y = −0.5227X^2^+4.561X+26	4.36	0.003	0.8150
GSH-Px	Y = −9.957X^2^+129.6X+433.6	6.51	0.026	0.8439
T-SOD	Y = −28.65X^2^+286X+57.31	4.99	0.005	0.7001
Crude ash	Y = −1.738X^2^+17.82X−14.31	5.13	0.001	0.7668

1) Quadratic polynomial: Y = *β*_0_+*β*_1_×X+*β*_2_×X^2^, where Y is the response variable, X is the total dietary VB1 concentration (mg/kg), *β*_0_ is the intercept, and *β*_1_ and *β*_2_ are the linear and quadratic coefficients, respectively; VB1 for the maximum response is calculated as −*β*_1_÷(2×*β*_2_).

GSH-Px, glutathione peroxidase; T-SOD, total superoxide dismutase.

## SUPPLEMENTARY MATERIAL

**Supplement 1.** Effects of dietary vitamin B1 on laying performance, egg quality, serum biochemicals, antioxidant capacity, and nutrient digestion ability of geese.

## References

[1] Shi ZD, Tian YB, Wu W, Wang ZY (2008). Controlling reproductive seasonality in the geese: a review. Worlds Poult Sci J.

[2] Yang YZ, Yao Y, Cao ZF, Gu TT, Xu Q, Chen GH (2019). Histological characteristics of follicles and reproductive hormone secretion during ovarian follicle development in laying geese. Poult Sci.

[3] Zhang Y, Chen ZY, An C (2021). Effect of active immunization with recombinant-derived goose INH-α, AMH, and PRL fusion protein on broodiness onset and egg production in geese (Anser cygnoides). Poult Sci.

[4] Brahman PK, Dar RA, Pitre KS (2013). DNA-functionalized electrochemical biosensor for detection of vitamin B1 using electrochemically treated multiwalled carbon nanotube paste electrode by voltammetric methods. Sens Actuators B Chem.

[5] Bunik VI, Aleshin VA, Zhou X, Krishnan S, Karlsson A (2020). Regulation of thiamine (vitamin B1)-dependent metabolism in mammals by p53. Biochem Mosc.

[6] Manzetti S, Zhang J, van der Spoel D (2014). Thiamin function, metabolism, uptake, and transport. Biochemistry.

[7] Mkrtchyan G, Aleshin V, Parkhomenko Y (2015). Molecular mechanisms of the non-coenzyme action of thiamin in brain: biochemical, structural and pathway analysis. Sci Rep.

[8] Singleton CK, Martin PR (2001). Molecular mechanisms of thiamine utilization. Curr Mol Med.

[9] Tylicki A, Siemieniuk M (2011). Thiamine and its derivatives in the regulation of cell metabolism. Postepy Hig Med Dosw.

[10] Coward KH, Morgan BGE, Waller L (1942). The influence of a deficiency of vitamin B1 and of riboflavin on the reproduction of the rat. J Physiol.

[11] Yazlık M, Olğaç KT, Çolakoğlu HE, Kaya U, Yildirim MM, Baş B (2021). Effects of vitamin B1, vitamin E and selenium on pregnancy and blood metabolites profile during non-breeding season and early prediction of pregnancy by thermographic monitoring in Merino ewes. Indian J Anim Sci.

[12] Olkowski AA, Classen HL (1996). The study of thiamine requirement in broiler chickens. Int J Vitam Nutr Res.

[13] Chen W, Fouad AM, Ruan D, Wang S, Xia WG, Zheng CT (2018). Effects of dietary thiamine supplementation on performance, egg quality, and antioxidant-related enzymes in Chinese egg-laying ducks. J Anim Plant Sci.

[14] Wang J, Wang BW, Ge WH (2013). Effects of vitamin B1 level on growth performance, activity of gastrointestinal digestive enzymes and TPK1 gene expression in liver of brood and finishing geese and study of correlation. Chin J Anim Vet Sci.

[15] Dale N (1994). National Research Council nutrient requirements of poultry - ninth revised edition (1994). J Appl Poult Res.

[16] Kleindorfer S, Krupka MA, Katsis AC, Frigerio D, Common LK (2024). Aggressiveness predicts dominance rank in greylag geese: mirror tests and agonistic interactions. R Soc Open Sci.

[17] Hao J, Zhang B, Wang B, Zhang M, Fan W, Li W (2023). Effects of dietary vitamin K3 supplementation on production performance, egg quality, vitamin K-dependent proteins, and antioxidant properties in breeding geese during the laying period. Poult Sci.

[18] Baur FJ, Ensminger LG (1977). The Association of Official Analytical Chemists (AOAC). J Am Oil Chem Soc.

[19] Bess F, Vieira SL, Favero A, Cruz RA, Nascimento PC (2012). Dietary iron effects on broiler breeder performance and egg iron contents. Anim Feed Sci Technol.

[20] Taschetto D, Vieira SL, Angel CR (2017). Iron requirements of broiler breeder hens. Poult Sci.

[21] Suzuki T, Ichinoe K, Ishijima Y, Masushige S (1967). Effects of some novel thiamine derivatives in chickens. Jpn Poult Sci.

[22] Remus JC, Firman JD (1990). Effect of thiamin deficiency on energy metabolites in the turkey. J Nutr Biochem.

[23] Olkowski AA, Classen HL (1999). The effects of maternal thiamine nutrition on thiamine status of the offspring in broiler chickens. Int J Vitam Nutr Res.

[24] Hegsted DM, Rao MN (1945). Nutritional studies with the duck: II. pyridoxine deficiency: one figure. J Nutr.

[25] Li Q, Zhang M, Wang B, Fan W, Kong M, Wang C (2023). Effects of dietary vitamin B1 supplemental level on reproductive performance, serum reproductive hormone indices, intestinal tissue morphology and cecal flora structure of breeding geese during laying period. Chin J Anim Nutr.

[26] Polin D, Wynosky ER, Porter CC (1963). Amprolium: XI. studies on the absorption of amprolium and thiamine in laying hens. Poult Sci.

[27] Ross JP, Honeyfield DC, Brown SB (2009). Gizzard shad thiaminase activity and its effect on the thiamine status of captive American alligators Alligator mississippiensis. J Aquat Anim Health.

[28] Elwinger K, Fisher C, Jeroch H, Sauveur B, Tiller H, Whitehead CC (2016). A brief history of poultry nutrition over the last hundred years. Worlds Poult Sci J.

[29] Zhang B, Tang J, Wu YB (2021). Effects of riboflavin deficiency on the lipid metabolism of duck breeders and duck embryos. Poult Sci.

[30] Daghir NJ (1976). Vitamin B6 in poultry nutrition A review. Worlds Poult Sci J.

[31] King’ori AM (2011). Review of the factors that influence egg fertility and hatchabilty in poultry. Int J Poult Sci.

[32] Zang H, Zhang K, Ding X, Bai S, Hernández JM, Yao B (2011). Effects of different dietary vitamin combinations on the egg quality and vitamin deposition in the whole egg of laying hens. Braz J Poult Sci.

[33] Ellis NR, Miller D, Titus HW, Byerly TC (1933). Effect of diet on egg composition: III. the relation of diet to the vitamin B and the vitamin G vontent of eggs, together with observations on the vitamin A content. J Nutr.

[34] Janist N, Srichana P, Asawakarn T, Kijparkorn S (2019). Effect of supplementing the laying hen diets with choline, folic acid, and vitamin B12 on production performance, egg quality, and yolk phospholipid. Livest Sci.

[35] Ojelade AYP (2016). Effects of proprietary vitamin-mineral premixes and housing systems on laying chickens egg production and quality indices [dissertation].

[36] Mandal AB, Jalaludeen A, Churchil RR, Baéza E (2022). Feeding and nutrient requirements of ducks. Duck production and management strategies.

[37] Hamano Y (1999). Effects of thiamine and clenbuterol on body composition, plasma metabolites and hepatic oxygen consumption in broiler chicks. Br Poult Sci.

[38] Tang J, Wu Y, Zhang B (2022). Integrated liver proteomics and metabolomics identify metabolic pathways affected by pantothenic acid deficiency in Pekin ducks. Anim Nutr.

[39] Witten S, Aulrich K (2019). Exemplary calculations of native thiamine (vitamin B1) and riboflavin (vitamin B2) contents in common cereal-based diets for monogastric animals. Org Agric.

[40] Lala V, Zubair M, Minter DA (2023). Liver function tests [Internet]. StatPearls.

[41] Shibuya M, Nagata K, Okada M (1982). Effect of pyridoxine deficiency on activities and amounts of aspartate aminotransferase isozymes in rat tissues. J Biochem.

[42] Tejpal CS, Chatterjee NS, Elavarasan K (2017). Dietary supplementation of thiamine and pyridoxine-loaded vanillic acid-grafted chitosan microspheres enhances growth performance, metabolic and immune responses in experimental rats. Int J Biol Macromol.

[43] Ueda T, Arakawa M, Kotake Y (1967). Changes of aspartate aminotransferase and alanine aminotransferase activity in vitamin B6 deficient rat. Nagoya J Med Sci.

[44] Salvatori G, Mondeì V, Piersigilli F (2016). Thiamine deficiency in a developed country. J Parenter Enteral Nutr.

[45] Wen LM, Feng L, Jiang WD (2016). Thiamin deficiency induces impaired fish gill immune responses, tight junction protein expression and antioxidant capacity: roles of the NF-κB, TOR, p38 MAPK and Nrf2 signaling molecules. Fish Shellfish Immunol.

[46] Ma Y, Wang C, Elmhadi M (2021). Thiamine ameliorates metabolic disorders induced by a long-term high-concentrate diet and promotes rumen epithelial development in goats. J Dairy Sci.

[47] Bunchasak C, Kachana S (2009). Dietary folate and vitamin B12 supplementation and consequent vitamin deposition in chicken eggs. Trop Anim Health Prod.

[48] Thonney ML, Palhof BA, DeCarlo MR (1985). Sources of variation of dry matter digestibility measured by the acid insoluble ash marker. J Dairy Sci.

[49] Mohseni M, Ghelichpour M, Hassani MHS, Pajand ZO, Vaghei RG (2023). Effects of dietary thiamine supplementation on growth performance, digestive enzymes’ activity, and biochemical parameters of Beluga, Huso huso, larvae. J Appl Ichthyol.

[50] Bokhorov O, Lazarov I, Bozhkov S (1984). Changes in the cAMP content in the intestinal and liver tissue and in 35S-thiamine transport in chickens fed a low-protein diet. Vet Med Nauki.

[51] Said HM, Ortiz A, Kumar CK, Chatterjee N, Dudeja PK, Rubin S (1999). Transport of thiamine in human intestine: mechanism and regulation in intestinal epithelial cell model Caco-2. Am J Physiol Cell Physiol.

[52] Aleshin VA, Mkrtchyan GV, Bunik VI (2019). Mechanisms of non-coenzyme action of thiamine: protein targets and medical significance. Biochem Mosc.

[53] Ang CYW, Jung HC, Benoff FH, Charles OW (1984). Effect of feeding three levels of riboflavin, niacin and vitamin B6 to male chickens on the nutrient composition of broiler breast meat. J Food Sci.

[54] Standardization Administration of China (2017). Determination of the contents of calcium, copper, iron, magnesium, manganese, potassium, sodium and zinc in feeds—method using atomic absorption spectrometry. Stansdard No. GB/T 13885-2017.

