# Supplementary material for: Optimal dietary vitamin B1 content enhanced egg production, eggshell thickness, and serum antioxidant status in breeder geese
Source: Anim Biosci. 2025 Feb 27;38(8):1746–55. doi: 10.5713/ab.24.0751 (PMC12229916; doi:10.5713/ab.24.0751)
Supplement: Supplementary file 1 [file ab-24-0751-Supplementary-1.pdf]

Supplementary material

Effects of dietary vitamin B1 on laying performance, egg quality, serum biochemicals, antioxidant capacity, and nutrient digestion ability of geese

Lin *Dai* [1,a], Bao Wei *Wang* [1*], Qian *Li* [1,a], Ming Ai *Zhang* [1], Jing *Zhang* [2], Bin *Yue* [3], Min *Kong* [4], Bing Han *Wang* [5], and Wen Lei *Fan* [1*]

[*]**Corresponding Author: Bao Wei Wang**

Tel: +86 0532-58957780, **E-mail**: wangbw@qau.edu.cn

Wen Lei Fan

Tel: +86 0532-58977771, **E-mail**: fanwenlei@qau.edu.cn,

[1] College of Animal Science and Technology, Qingdao Agricultural University, Qingdao 266109, China

[2] College of Food Science and Engineering, Qingdao Agricultural University, Qingdao 266109, China

[3] College of Science and Information Science, Qingdao Agricultural University, Qingdao 266109, China

[4] Institute of high-quality waterfowl, Qingdao Agricultural University, Qingdao 266109, China

[5] Qingdao Huihe Biotechnology Co., Ltd, Qingdao 266109, China

[a] These authors contributed equally to this work.

Egg quality

Egg quality was assessed using the DET-6000 Egg Analyzer, an automated instrument ensuring accuracy and efficiency. The procedures were as follows:

1) Eggshell Strength: Eggs were placed on the measurement platform, and pressure was applied gradually until the shell broke. The maximum pressure at breakage indicated the eggshell strength.

2) Eggshell Thickness: Thickness was measured at three points—the sharp end, blunt end, and middle—using a precise gauge. The average thickness was calculated from these measurements.

3) Albumen Height: The egg was cracked open, and the albumen was placed on the platform. A sensor measured the height of the highest point, which was recorded for analysis.

4) Yolk Color: A specialized colorimeter measured the yolk's color by comparing it to a standard color chart, generating a color grade.

5) Haugh Unit (HU): The Haugh Unit was calculated using the measured egg weight and albumen height according to a specific formula.

Biochemical -Related Parameters

Frozen serum samples were thawed on ice and then centrifuged at 5 000×*g* for 5 minutes. Concentrations of triglycerides (**TG**, #A110-1-1), total cholesterol (**T-CHO**, #A111-1-1), albumin (**ALB**, #A028-2-1), total protein (**TP**, #A045-2-2), and the activities of aspartate transaminase (**AST**, #C010-2-1) and alanine transaminase (**ALT**, #C009-2-1) were measured using kits from Nanjing Jiancheng Bioengineering Institute and Beijing Leagene Biotechnology.

Serum TG was measured using the GPO-PAP enzyme method. Samples were mixed with the enzyme reagent (1:10 ratio) and incubated at 37°C for 10 minutes. Absorbance was recorded at 500 nm using a microplate reader (Model 680, BIO-RAD, Japan), and TG content was calculated using the formula provided.

ALB concentration was determined using the bromocresol green method. Samples were mixed with the color reagent (1:10 ratio) and allowed to stand at room temperature for 10 minutes. Absorbance was measured at 628 nm, and the ALB content was calculated accordingly.

TP was measured using the Coomassie Brilliant Blue method. Samples were mixed with the color reagent (1:60 ratio) and incubated at room temperature for 10 minutes. Absorbance was recorded at 595 nm, and TP content was calculated using the specified formula.

AST activity was determined using the Reitman-Frankel method. Samples were mixed with the AST substrate (1:4 ratio) and incubated at 37°C for 30 minutes. After adding 2,4-dinitrophenylhydrazine, the mixture was further incubated at 37°C for 20 minutes, followed by the addition of 0.4 mol/L sodium hydroxide. After standing at room temperature for 15 minutes, absorbance was measured at 510 nm. A standard curve with sodium pyruvate was used to calculate AST activity.

ALT activity was also determined using the Reitman-Frankel method. Samples were mixed with the ALT substrate (1:4 ratio) and incubated at 37°C for 30 minutes. After adding 2,4-dinitrophenylhydrazine and further incubation for 20 minutes, 0.4 mol/L sodium hydroxide was added. The mixture was allowed to stand at room temperature for 15 minutes, and absorbance was measured at 505 nm. ALT activity was calculated using a standard curve

with sodium pyruvate.

Antioxidation-Related Parameters

Frozen serum samples were thawed on ice and then centrifuged at 5 000×*g* for 5 min. Malondialdehyde (**MDA**, #A003-1-2), total superoxide dismutase (**T-SOD**, #A001-3-2), total antioxidant capacity (**T-AOC**, #A015-3-1), and glutathione peroxidase (**GSH-Px,** #A005-1-2) were determined using commercial kits (Nanjing Jiancheng Bioengineering Institute, and Beijing Leagene Biotechnology). The methods for each analysis are detailed below:

Measured using the thiobarbituric acid (**TBA**) method. Samples were incubated with MDA standard reagents at 95°C for 40 minutes, then cooled on ice and centrifuged at 3,500×g for 10 minutes. A 200 μL aliquot of the supernatant was transferred to 96-well plates, and absorbance was recorded at 532 nm using a microplate reader (Model 680, BIO-RAD, Japan). MDA content was calculated based on the absorbance.

Determined using the xanthine oxidase method. SOD in the samples reduced nitrite production, which was induced by the xanthine oxidase system. Absorbance was measured at 550 nm, and T-SOD activity was calculated based on nitrite reduction.

Measured by the oxidation rate of glutathione (**GSH**). Samples were mixed with 1 mmol/L GSH solution, centrifuged, and the supernatant was combined with GSH-Px assay reagents. Absorbance was recorded at 412 nm, and GSH-Px activity was calculated using the resulting data.

Assessed using the ferric ion reducing antioxidant power (**FRAP**) method. A standard curve was prepared using known concentrations of FeSO4. For the assay, 180 μL of FRAP reagent and 5 μL of serum or standard were added to each well of a microplate, incubated at

37°C for 5 minutes, and absorbance was measured at 593 nm. T-AOC levels were calculated by comparing sample absorbance to the standard curve.
